# Antibody–Drug Conjugates in Contemporary Oncology: A Clinical Perspective on Dose Optimization, Sequencing, and Combination Strategies

**DOI:** 10.3390/cancers18162652

**Published:** 2026-08-17

**Authors:** Alexander Philipovskiy, Scott Shurmur, Muhammad Bilal Abid

**Affiliations:** 1 Division of Hematology/Oncology, Department of Internal Medicine, Texas Tech University Health Sciences Center and UMC/TLC2 Cancer Center, 602 Indiana Ave., Lubbock, TX 79415, USA; 2 Department of Internal Medicine, Texas Tech University Health Sciences Center, 3601 4th Street, Lubbock, TX 79430, USA

**Keywords:** antibody–drug conjugates, dose optimization, sequencing, combination strategies, biomarkers, resistance, interstitial lung disease, linker, payload, translational oncology

## Abstract

Antibody–drug conjugates (ADCs) have matured from a technically challenging concept into an established therapeutic platform across hematologic and solid malignancies. By linking a monoclonal antibody to a potent cytotoxic payload, ADCs seek to increase tumor-directed drug delivery while limiting systemic exposure; however, clinical performance depends on much more than target expression. Target accessibility, antigen density and heterogeneity, internalization, intracellular trafficking, linker stability, payload properties, drug-to-antibody ratio, bystander effect, tumor penetration, and host-tissue handling jointly shape efficacy and toxicity. This review provides a clinician-oriented assessment of the contemporary ADC landscape and the principal biologic and pharmacologic determinants of benefit. We discuss signature toxicities, including interstitial lung disease and ocular injury, and summarize evolving mechanisms of resistance and biomarker development. Particular emphasis is placed on three translational problems that are becoming increasingly important as ADCs move into earlier lines and overlapping disease settings: dose optimization beyond the maximum tolerated dose, sequencing across targets and payload classes, and biologically rational combination strategies. We also present a conceptual Sequential Tumor Attack Model as a hypothesis-generating framework for tumor-access priming, ADC-mediated cytotoxic injury, and immune amplification, while explicitly recognizing that the complete model has not been clinically validated. The future impact of ADCs will depend not only on next-generation constructs, but also on rigorous evidence for how these agents should be dosed, ordered, and combined in practice.

## Simple Summary

Antibody–drug conjugates are cancer treatments designed to deliver potent cytotoxic drugs more selectively to tumor cells by linking a tumor-directed antibody to a therapeutic payload. This review examines the structural and biologic features that determine clinical activity, including target selection, linker stability, payload class, drug-to-antibody ratio, tumor penetration, biomarkers, toxicity, and resistance. Particular attention is given to the practical questions emerging as multiple ADCs enter the same malignancies and earlier treatment settings: how to select a biologically efficient dose, how to sequence agents with overlapping targets or payloads, and how to distinguish rational combinations from combinations that merely add toxicity. The next phase of ADC development will require not only improved molecular engineering, but also a more disciplined clinical framework for selecting, dosing, sequencing, and combining these agents.

## 1. Introduction

More than a century ago, Paul Ehrlich introduced the idea of selective therapy, envisioning agents that could specifically seek out diseased cells while sparing normal tissues. This “magic bullet” concept became one of the foundational ideas of modern targeted therapy. Antibody–drug conjugates embody this principle in a particularly direct way: a monoclonal antibody provides specificity, while a highly potent cytotoxic payload provides cell-killing capacity. In principle, ADCs offer a means of restoring selectivity to chemotherapy by converting it from a broadly distributed systemic intervention into a biologically guided targeted therapy [1].

The path from concept to clinical reality, however, has been long and technically difficult. Early ADC development was limited not only by unstable linkers, inconsistent conjugation chemistry, poor intracellular trafficking, and a narrow therapeutic index, but also by an incomplete understanding of which cell-surface proteins could serve as clinically useful targets. Many of the antigens that now anchor the modern ADC pipeline were discovered years or even decades earlier, yet their value as therapeutic entry points was not initially obvious. In solid tumors especially, the field lacked a sufficiently mature catalog of targets that combined tumor-enriched expression, membrane accessibility, efficient internalization, and an acceptable normal-tissue distribution. As a result, early target selection was often empiric, biologically incomplete, or overly optimistic, and many candidate targets ultimately failed to support sustained tumor selectivity. The later emergence of clinically relevant targets such as TROP2, CDH6, PTK7, B7-H3, B7-H4, and selected claudins illustrates how much ADC progress depended not only on better linker and payload chemistry, but also on a deeper biologic understanding of cell-surface architecture. In that sense, the slow maturation of the ADC field reflected both technological limitations and a delayed recognition of the target landscape required for successful selective payload delivery [2,3,4,5].

That situation has changed substantially. Modern ADCs now occupy a central role in several disease settings, particularly in breast cancer, urothelial carcinoma, ovarian cancer, non-small cell lung cancer, and hematologic malignancies. For a clinician-oriented view of the field, several recent regulatory milestones are especially important. *Trastuzumab deruxtecan* received accelerated approval in April 2024 for previously treated unresectable or metastatic HER2-positive solid tumors, providing the clearest current proof of concept for a tumor-agnostic ADC strategy. Sacituzumab govitecan is established in metastatic triple-negative breast cancer and in unresectable or metastatic HR-positive, HER2-negative breast cancer after endocrine-based therapy and additional systemic treatment. Datopotamab deruxtecan entered clinical practice for unresectable or metastatic HR-positive, HER2-negative breast cancer after prior endocrine therapy and chemotherapy, and subsequently expanded into EGFR-mutated non-small cell lung cancer after prior EGFR-directed therapy and platinum-based chemotherapy. Telisotuzumab vedotin was approved for previously treated non-squamous non-small cell lung cancer with high c-Met protein overexpression, and belantamab mafodotin returned for relapsed or refractory multiple myeloma in combination with bortezomib and dexamethasone. ADCs are no longer isolated proof-of-principle products. Instead, they represent a clinically validated drug-development platform with expanding relevance across oncology [6,7,8,9,10,11,12,13,14,15,16].

Importantly, ADC development is also beginning to intersect with a broader precision-oncology vision. While most ADCs remain developed and used within histology-specific treatment algorithms, some recent advances suggest that target expression may, in selected contexts, transcend tissue of origin. This possibility is highly relevant to the future of the field. ADCs may ultimately help bridge the gap between conventional organ-based oncology and a more biomarker-defined, tumor-agnostic therapeutic model [17].

This review is not intended to provide an exhaustive catalog of all ADC trials or investigational constructs. Instead, it focuses on the contemporary clinical and translational questions that have become increasingly important as multiple ADCs enter overlapping disease settings: how these agents should be dosed, how they should be sequenced, and which combination strategies are supported by biologic rationale and clinical evidence. Toxicity, biomarkers, resistance, and next-generation architecture are discussed selectively where they directly inform these decisions [18].

## 2. Current Clinical Landscape of ADCs

ADCs are now established in both hematologic and solid tumors. In hematologic malignancies, lineage-specific targets and favorable target biology have made ADC development relatively successful. These diseases often provide high antigen density, efficient internalization, and fewer physical barriers to drug delivery. Consequently, hematologic cancers were among the earliest diseases in which ADCs demonstrated substantial clinical utility [7,10].

Solid tumors proved more difficult. They introduce challenges that include antigen heterogeneity, stromal barriers, variable internalization, nonuniform vascular access, and greater potential for low-level target expression in critical normal tissues. Despite these barriers, the last decade has seen major clinical success. HER2-directed ADCs transformed the treatment landscape in breast cancer and later expanded into additional tumor types. TROP2-directed ADCs validated a broadly relevant solid-tumor target. Nectin-4-directed therapy has become central to urothelial cancer treatment. Folate receptor alpha has emerged as a clinically relevant biomarker-defined target in ovarian cancer. c-Met-directed therapy further illustrates the growing diversity of ADC target classes in lung cancer [6,8,9,14].

The clinical role of ADCs is also changing in terms of treatment timing. For many years, ADC development was concentrated primarily in heavily pretreated metastatic disease, where proof of activity and manageable toxicity were the dominant objectives. That pattern is now clearly beginning to change. ADCs are increasingly being studied and, in selected settings, approved earlier in the treatment course, including first-line metastatic combinations, residual-disease strategies, and early-stage disease. Trastuzumab deruxtecan is a particularly important example of this transition. Although its earlier development was centered in metastatic disease, it has now moved into HER2-positive early breast cancer strategies, including neoadjuvant and post-neoadjuvant settings. In addition, trastuzumab deruxtecan in combination with pertuzumab has entered the first-line treatment landscape for unresectable or metastatic HER2-positive breast cancer. These milestones are notable not only for breast cancer practice but also because they signal that ADCs are no longer confined to salvage therapy and are beginning to move into settings historically dominated by conventional chemotherapy [19,20,21,22,23,24].

Contemporary ADC development is therefore no longer defined simply by the number of new constructs entering trials. The field is now confronting overlapping targets, recurrent use of the same payload classes, movement into curative-intent settings, and approved combinations that compete with established chemotherapy-based regimens. These developments raise questions about treatment duration, cumulative toxicity, cross-resistance, and preservation of later options that cannot be answered by single-agent response rates alone [19,21,22,23,24].

Selected clinically established ADCs and representative design features are summarized in Table 1.

## 3. Determinants of ADC Efficacy

### 3.1. Target Selection and Antigen Biology

Target selection remains foundational to ADC success, but target expression alone does not guarantee effective payload delivery. After intravenous administration, ADCs must remain sufficiently stable in circulation, exit the vasculature, traverse the tumor interstitium, bind accessible antigen on tumor cells, undergo productive internalization, and release payload intracellularly. Each of these steps may be limited by tumor architecture. In solid tumors, abnormal vasculature, elevated interstitial pressure, dense extracellular matrix, and heterogeneous antigen distribution can all impair effective ADC penetration. Conversely, normal tissues are not absolutely shielded from antibody exposure, since IgG-based therapeutics can distribute into interstitial compartments, although usually at lower and slower levels than in plasma. Thus, selectivity reflects not only target abundance, but also tissue accessibility, vascular permeability, stromal structure, and local cellular handling of the conjugate. In this context, the tumor microenvironment may itself become a source of resistance: Fcγ receptor-expressing macrophages and other stromal elements may sequester, process, or redirect ADCs before adequate delivery to tumor cells occurs. This possibility is especially relevant in desmoplastic tumors, where impaired penetration and microenvironmental trapping may reduce effective exposure despite retained target expression [4,40].

### 3.2. Internalization and Intracellular Trafficking

Following target engagement, the ADC must be internalized and trafficked to the intracellular compartments where linker cleavage or antibody degradation can release the active payload. This process varies substantially among targets. Some antigens bind efficiently but internalize poorly, whereas others may recycle or traffic along pathways that are less favorable for productive payload release. Thus, cell-surface binding does not necessarily translate into cytotoxic delivery [40].

The efficiency of lysosomal processing is especially important for many ADCs. Altered intracellular trafficking, impaired lysosomal function, or changes in endosomal sorting may all affect drug sensitivity. In this way, ADCs differ from many conventional targeted therapies: their activity depends not only on receptor engagement, but also on the downstream physical handling of the conjugate within the cell [40].

### 3.3. Linker Design and Payload Release

The linker is one of the most important determinants of ADC performance because it connects the antibody to the cytotoxic payload and helps determine when and where the payload is released. In broad terms, there are two common types of linkers: cleavable and noncleavable. Cleavable linkers are designed to break under specific conditions, such as low pH, protease activity, or intracellular reducing conditions, thereby releasing the payload more readily once the ADC reaches the tumor cell or tumor microenvironment. Examples include trastuzumab deruxtecan and brentuximab vedotin, both of which use cleavable linker strategies. Noncleavable linkers are generally more stable in circulation and usually require intracellular degradation of the antibody after internalization before the active payload can be released; trastuzumab emtansine is the classic example of this approach [41,42,43].

Each strategy has potential advantages and trade-offs. Cleavable linkers may support more efficient payload release and stronger bystander effect, which can be useful in heterogeneous solid tumors. Noncleavable linkers may provide greater plasma stability and more controlled intracellular catabolism, which in some settings may reduce off-tumor exposure. This balance is central to therapeutic index. An overly labile linker may increase systemic toxicity, while an overly stable linker may reduce efficacy. Linker design therefore represents not only a chemical choice, but also a biologic and pharmacologic strategy [41,42,43].

### 3.4. Payload Class, Potency, and Bystander Effect

Most approved ADCs employ microtubule inhibitors or topoisomerase I inhibitors as payloads, but these are not the only payload classes that have been used in ADC development. Other important payload groups include DNA-damaging agents such as calicheamicin and pyrrolobenzodiazepine dimers, as well as newer experimental payloads such as transcriptional inhibitors, immune-stimulating agents, and other nontraditional warheads. Microtubule inhibitors and topoisomerase I inhibitors remain the dominant clinically validated classes because they have demonstrated substantial antitumor activity, but they are also associated with recognizable toxicity patterns and potential resistance mechanisms. The goal is not simply to maximize potency. A payload that is too potent, too unstable, or too broadly permeable may damage normal tissues and erode the very selectivity that the ADC seeks to create [44].

The dominance of microtubule inhibitors and topoisomerase I inhibitors likely reflects not a single chemical advantage, but a favorable overall balance of properties. These payloads are potent enough to remain effective even though only a small fraction of the administered ADC ultimately reaches the tumor cell, yet they can also be chemically modified, conjugated, released intracellularly, and still retain meaningful biologic activity. In addition, their pharmacology has proved sufficiently predictable to support repeated clinical dosing. By contrast, some alternative payloads may be so potent or so difficult to control that even modest off-target release can narrow the therapeutic window. In this sense, the popularity of these payload classes likely reflects a practical balance among ultrapotency, linker compatibility, intracellular releasability, and tolerability rather than simple tradition alone [44,45].

The bystander effect is especially relevant in heterogeneous solid tumors. When the released payload is membrane permeable, it may diffuse into neighboring cells that express lower levels of the target or lack it altogether. This can broaden efficacy in tumors with patchy antigen distribution. However, bystander killing may also increase collateral damage in normal tissues if drug release occurs outside the intended tumor microenvironment. The bystander effect should therefore be viewed as a controllable design feature rather than an unqualified advantage [46,47].

### 3.5. Drug-to-Antibody Ratio and Product Homogeneity

Drug-to-antibody ratio (DAR) is an important determinant of ADC behavior because it influences how much payload is delivered with each antibody molecule, but more payload does not automatically translate into better clinical performance. In principle, a higher DAR may increase tumor-cell drug delivery and, in selected settings, may contribute to greater efficacy. The comparison between trastuzumab deruxtecan and trastuzumab emtansine is often cited as a practical example that a higher-DAR design can be associated with stronger clinical activity, particularly when paired with a cleavable linker and a membrane-permeable payload. However, a higher DAR also increases the risk of altering the behavior of the antibody scaffold, accelerating clearance, increasing aggregation, and narrowing the therapeutic window. Conversely, a lower-DAR ADC can still be highly effective when other properties are favorable, including target biology, linker design, payload potency, bystander effect, and combination partner selection, as illustrated by enfortumab vedotin. For this reason, DAR should be viewed not as a simple “more is better” variable, but as one component of a broader design balance among potency, pharmacokinetics, stability, and tolerability. Historically, stochastic conjugation produced highly heterogeneous ADC mixtures, complicating development and interpretation. Newer site-specific conjugation technologies aim to improve homogeneity and produce more predictable pharmacologic behavior [45,48,49].

## 4. Toxicity as a Central Development Challenge

The progress of ADCs has made clear that toxicity is not an incidental feature of the platform but one of its defining developmental constraints. Some adverse events reflect target expression in normal tissues, whereas others are driven primarily by linker instability, payload biology, off-tumor uptake, Fc-mediated handling, or systemic exposure to released payload. In practice, most clinically important toxicities likely reflect a combination of these mechanisms. For a clinician-facing review, it is useful to distinguish toxicities that require urgent recognition and treatment interruption from those that require structured surveillance and co-management [50,51,52,53,54].

Interstitial lung disease (ILD) and pneumonitis have emerged as signature toxicities of several topoisomerase I inhibitor–based ADCs, particularly deruxtecan-containing agents. With trastuzumab deruxtecan, ILD/pneumonitis requires active symptom surveillance, prompt evaluation of new respiratory symptoms, treatment interruption for suspected events, and permanent discontinuation for confirmed Grade 2 or higher toxicity. Current prescribing information reports ILD/pneumonitis in approximately 12% of patients treated with trastuzumab deruxtecan 5.4 mg/kg across breast cancer, HER2-mutant non-small cell lung cancer, and other solid tumors, with a median time to onset of 5.5 months and fatal events in approximately 0.9% of patients. Datopotamab deruxtecan carries a similar warning, with recommendations to monitor for cough, dyspnea, fever, and other new or worsening respiratory symptoms, to withhold treatment when ILD is suspected, and to permanently discontinue therapy for confirmed Grade 2 or higher ILD/pneumonitis [50,51,53,54].

The biologic basis of ADC-associated ILD remains incompletely defined and is likely multifactorial. Proposed mechanisms include Fcγ receptor-mediated uptake of intact ADCs by alveolar or interstitial macrophages, intracellular processing with local payload release, premature systemic deconjugation, and nonspecific uptake by pulmonary cells. Linker stability, payload permeability, cumulative exposure, and pre-existing pulmonary susceptibility may also contribute. The occurrence of ILD across several topoisomerase I inhibitor ADCs raises the possibility of a payload- or platform-related effect, but risk is not uniform across agents and no single mechanism has been established [54].

The practical importance of early recognition cannot be overstated. Grade 1 ILD/pneumonitis is, by definition, asymptomatic and detected radiographically or clinically without symptoms. This is the critical point at which treatment interruption, diagnostic clarification, and early corticosteroid consideration may prevent progression to symptomatic disease. Once symptoms develop, most labels require permanent discontinuation. Therefore, clinicians should educate patients from the first cycle to report even mild cough, exertional dyspnea, unexplained fever, or reduced exercise tolerance. A low threshold for chest CT imaging is warranted, particularly because early symptoms may be misattributed to infection, prior radiation, underlying lung disease, or disease progression [50,53].

Pulmonary toxicity should also be viewed as a platform-level concern rather than as an isolated property of one approved drug. The investigational CDH6-directed ADC raludotatug deruxtecan further illustrates this point. In published early clinical experience, treatment-related ILD/pneumonitis has been observed, including Grade 2 events in the ovarian cancer subgroup from phase 1 testing and additional events reported in later dose-optimization cohorts. These data do not establish identical risk across all deruxtecan-based ADCs, but they reinforce the need for pulmonary vigilance during development and clinical use [50,51].

Ocular toxicity represents another clinically important ADC toxicity domain. Belantamab mafodotin is the clearest example, with corneal epithelial changes sufficiently frequent and clinically consequential to require structured ophthalmologic monitoring. Current prescribing information includes a boxed warning for ocular toxicity, and regulatory approval materials report high rates of ocular toxicity in the pivotal relapsed or refractory multiple myeloma setting, with many patients requiring dose interruption, reduction, or discontinuation. Because ocular findings may precede symptoms, baseline and serial ophthalmologic examinations should be considered part of treatment delivery rather than optional supportive care [15,52,54].

Datopotamab deruxtecan is also associated with ocular adverse reactions, including dry eye, keratitis, blepharitis, meibomian gland dysfunction, conjunctivitis, increased lacrimation, and blurred vision. The prescribing information recommends prophylactic preservative-free lubricating eye drops, avoidance of contact lenses unless directed by an eye care professional, referral for new or worsening ocular symptoms, and regular ophthalmologic assessment during treatment. For practicing oncologists, the key point is that ocular toxicity should not be managed as a minor symptomatic adverse event alone; it requires anticipatory counseling, early ophthalmology involvement, and dose modification when clinically indicated [51,52,53,54].

Other ADC-associated toxicities remain highly relevant, including myelosuppression, neuropathy, stomatitis, nausea, diarrhea, fatigue, infusion reactions, and hepatotoxicity. These adverse events may appear more familiar to oncologists because they overlap with conventional cytotoxic therapy, but their timing, reversibility, and relationship to cumulative exposure may differ by ADC construct, payload class, and schedule. For example, microtubule inhibitor–based ADCs are commonly associated with neuropathy and myelosuppression, whereas topoisomerase I inhibitor–based ADCs more often raise concern for gastrointestinal toxicity, stomatitis, myelosuppression, and ILD/pneumonitis. These patterns are clinically useful but should not be treated as rigid class rules [51,53,54].

A practical framework is therefore to divide ADC toxicity into three clinically actionable categories. The first includes toxicities requiring urgent exclusion and treatment interruption, particularly suspected ILD or pneumonitis. The second includes toxicities requiring structured co-management and surveillance, such as keratopathy and ocular surface injury. The third includes common but still clinically important toxicities such as myelosuppression, neuropathy, stomatitis, gastrointestinal toxicity, fatigue, and infusion reactions. This framework better reflects real-world decision making than a simple adverse-event list and emphasizes a central principle of contemporary ADC practice: the success of these agents depends not only on selective payload delivery, but also on early detection, patient education, and disciplined management of construct-specific toxicities [51,53,54].

## 5. Mechanisms of Resistance

Resistance to ADC therapy can arise at multiple levels. The most obvious mechanism is antigen loss or downregulation. Under treatment pressure, tumors may reduce expression of the target or select for antigen-low subclones. This is particularly relevant in heterogeneous tumors and may become increasingly important as ADCs move earlier in treatment sequences [55,56,57,58,59].

Resistance may also develop downstream of target binding. Changes in endocytosis, intracellular trafficking, lysosomal function, or linker cleavage may reduce effective payload release. Upregulation of efflux transporters can lower intracellular exposure to certain membrane-permeable payloads. Tumor cells may further resist payload action through alterations in microtubules, DNA damage response, apoptotic threshold, or broader changes in cell state [55,58,59].

An additional practical question is cross-resistance. As multiple ADCs enter the same disease space, it becomes increasingly important to understand whether resistance to one ADC predicts reduced sensitivity to another, particularly when agents share a target, payload class, or intracellular processing pathway. This area remains incompletely defined and has major implications for future sequencing strategies [55,58,59].

## 6. Biomarkers and Patient Selection

Biomarker development remains one of the major unresolved challenges in ADC therapy. Immunohistochemistry has been the most common method for patient selection, but static target expression captures only one component of ADC sensitivity. It does not directly measure target accessibility, internalization, intracellular trafficking, lysosomal processing, payload susceptibility, or the extent to which the bystander effect may compensate for heterogeneous expression [56,57,60,61].

Recent clinical experience has challenged traditional binary biomarker thinking. Activity in HER2-low and other lower-expression settings demonstrates that clinically meaningful benefit may extend beyond tumors with uniformly high antigen expression. At the same time, lowering thresholds indiscriminately can expose patients to toxicity without sufficient probability of benefit; quantitative and context-specific interpretation remains necessary [28,29,56].

Antigen expression is also dynamic. Target levels may change during treatment, after exposure to target-directed therapy, or during metastatic progression, and discordance may occur between the primary tumor and metastatic sites or among separate lesions. These temporal and spatial changes may influence sequential ADC activity and support reassessment of target expression when clinically feasible, particularly after treatment with an ADC directed against the same antigen [55,56,57,60].

Liquid biopsy may complement tissue assessment by identifying genomic evolution, emerging resistant clones, or alterations in payload-response pathways. However, circulating tumor DNA does not directly measure surface antigen density, internalization, or whole-body target accessibility. Molecular imaging approaches such as immuno-PET may eventually provide noninvasive assessment of target distribution and interlesional heterogeneity, although these methods remain investigational and require prospective validation [60,62,63,64,65].

Predictive biomarker development should therefore extend beyond antigen expression to include target persistence, endosomal and lysosomal processing, drug-efflux pathways, DNA-damage response, apoptotic competence, and microenvironmental factors that influence penetration or nonproductive sequestration. Integrated tissue, blood, and imaging biomarkers may ultimately be required to predict both initial benefit and resistance [55,58,59,60].

## 7. ADCs and the Emerging Tumor-Agnostic Paradigm

One of the most intriguing recent developments in oncology is the gradual movement from histology-based treatment toward biomarker-defined therapy. ADCs may become increasingly relevant to this shift. Because they are built around the principle of selective delivery to a biologically defined target, they are conceptually well suited to a tumor-agnostic strategy [28,29,66,67].

In practice, however, broad tumor-agnostic ADC therapy remains at an early stage. Most ADCs are still developed within conventional disease-specific programs, and the same target may behave differently across tumor types because of differences in antigen density, internalization, stromal context, trafficking biology, and toxicity tolerance. A target that is highly actionable in one tumor type may be far less useful in another, even when nominal expression is present [28,29,67].

Nevertheless, the broader direction is clear. ADCs increasingly support the idea that target biology, when sufficiently robust, may sometimes supersede tissue of origin as the primary organizing principle for treatment selection. This does not mean that histology will disappear from oncology decision making. Rather, it suggests that ADCs may help create a hybrid model in which both tissue context and actionable target expression guide therapy [28,29,67].

This theme is particularly important for the future of drug development. If ADCs can reliably function across multiple histologies defined by shared target biology, they may become a major vehicle for expanding precision oncology beyond small-molecule inhibitors and immune biomarkers. The success of that transition will depend on better biomarker assays, deeper biologic understanding of target behavior across cancers, and careful management of toxicity in diverse patient populations [28,29,67].

## 8. Dose, Sequence, and Combination: The Translational Gaps Between Development and Practice

As ADCs mature from late-line agents into platform therapies used across multiple disease settings, one of the central unresolved questions is no longer whether they can work, but how they should be used most rationally. The first generation of ADC approvals largely emerged from heavily pretreated populations in which dose intensity and single-agent activity were the dominant developmental priorities. That framework is no longer sufficient. Once multiple ADCs become available within the same tumor type, clinicians are forced to confront a new set of practical questions: whether an ADC should be given at the highest tolerated dose or at the most pharmacologically efficient dose; whether one ADC can be used after another; whether switching target is more important than switching payload; and which partner therapies are likely to produce true synergy rather than overlapping toxicity.

### 8.1. Dose Optimization Beyond Conventional Oncology Logic

Dose optimization for ADCs remains underdeveloped relative to the complexity of the platform. Traditional oncology drug development often relies on escalation toward the maximum tolerated dose, but this paradigm may be poorly suited to ADCs, whose toxicities may be delayed, organ-specific, or driven by distinct behaviors of the antibody, linker, and payload components. In practice, the most useful dose may not be the highest dose that patients can briefly tolerate, but the dose that best balances exposure, sustained delivery, cumulative toxicity, and long-term treatment continuity. This issue becomes even more important when ADCs move into earlier-line, maintenance, or lower-burden disease settings, where tolerance for irreversible toxicity is substantially lower [3,40,49].

The problem is not purely academic. ADCs raise clinical pharmacology questions that are more complicated than those posed by either monoclonal antibodies or conventional chemotherapy alone. Weight-based versus fixed dosing, exposure-response relationships, intrinsic patient factors, delayed toxicity, and the contribution of released payload to overall pharmacology all complicate dose selection. These issues suggest that the next phase of ADC development will require a more deliberate dose-optimization strategy rather than simple inheritance of conventional cytotoxic development principles [3,40,49,68,69,70,71].

A more informative dose-selection strategy should distinguish exposure to intact ADC, total antibody, conjugated payload, and released payload, and should integrate delayed toxicity with duration of treatment rather than focusing only on acute dose-limiting events. Randomized dose-optimization cohorts, longitudinal pharmacokinetic–pharmacodynamic modeling, and patient-reported tolerability are particularly important when an ADC is intended for earlier-line or curative-intent use [3,40,49].

### 8.2. Sequencing in an Era of Multiple ADCs

Sequencing has rapidly become one of the most practical unresolved issues in ADC therapy. In diseases such as breast cancer, clinicians may now encounter several ADCs within a single treatment course, sometimes with overlapping targets, sometimes with distinct targets but related payload classes. Yet evidence to guide sequential use remains limited and is driven largely by retrospective data, disease-specific experience, and biologic inference rather than prospective sequencing trials [72,73,74,75].

Several nonexclusive questions arise in this setting. First, does resistance to one ADC meaningfully reduce the chance of benefit from a second ADC directed at a different target but carrying a similar payload? Second, if two ADCs share a target but differ in linker, drug-to-antibody ratio, and payload behavior, can one still be effective after failure of the other? Third, is the key determinant of sequential activity the target, the payload, the bystander effect, or the intracellular trafficking pathway? These questions are especially relevant in breast cancer, where sequential use of ADCs is already clinically real, but they will likely extend to other diseases as the ADC pipeline expands [72,73,74,75].

The sequencing problem is closely linked to the incomplete clinical mapping of cross-resistance. Retrospective breast cancer cohorts demonstrate that a second ADC can retain activity after prior ADC exposure, but the magnitude and duration of benefit are variable and are often more limited than with the first ADC. These findings do not support a simple rule that changing either target or payload will reliably overcome resistance. Prospective studies with paired biopsies and molecular assessment are needed to determine whether sequential benefit is driven primarily by target persistence, payload sensitivity, linker behavior, intracellular processing, or broader tumor evolution [72,73,74,75,76,77,78].

### 8.3. Combination Development: Biology Versus Enthusiasm

Combination development has proceeded rapidly, especially with immune checkpoint inhibitors, chemotherapy, and targeted agents. The rationale is attractive: ADCs may debulk disease selectively, induce immunogenic cell death, increase antigen release, and alter the tumor microenvironment in ways that sensitize tumors to immunotherapy or pathway inhibition. In some settings, combinations may also compensate for tumor heterogeneity or help prevent outgrowth of resistant clones. Practice-changing results with enfortumab vedotin plus pembrolizumab and trastuzumab deruxtecan plus pertuzumab demonstrate that ADC combinations can improve outcomes when the partner and disease context are appropriate; however, these successes do not establish a general principle that every ADC will benefit from combination therapy [19,21,22,79,80,81,82].

One especially important conceptual direction is to view ADCs not only as terminal cytotoxic agents, but also as biologic primers of the tumor microenvironment. In this framework, the first step is not indiscriminate inflammation, but tumor-selective vascular and immune priming. Such an approach could improve functional vascular access, reduce stromal resistance, and limit nonproductive sequestration by macrophages or other stromal elements before adequate delivery to tumor cells occurs. ADC-mediated tumor injury could then serve as a second step, creating antigen release, local danger signals, and conditions more favorable for adaptive immune activation. In principle, this sequence could convert incomplete direct tumor-cell killing into a broader immune-mediated antitumor response, particularly when paired with therapies that enhance antigen presentation, T-cell recruitment, or checkpoint release. Although this model remains partly theoretical, it provides a biologically coherent explanation for why some future ADC combinations may need to be designed not simply to add cytotoxic effect, but to improve tumor access and transform initial ADC-induced injury into more effective immune clearance. The components of this sequence are supported to varying degrees by preclinical and clinical observations, but the integrated sequence remains hypothetical [17,22,47].

**Figure 1:**
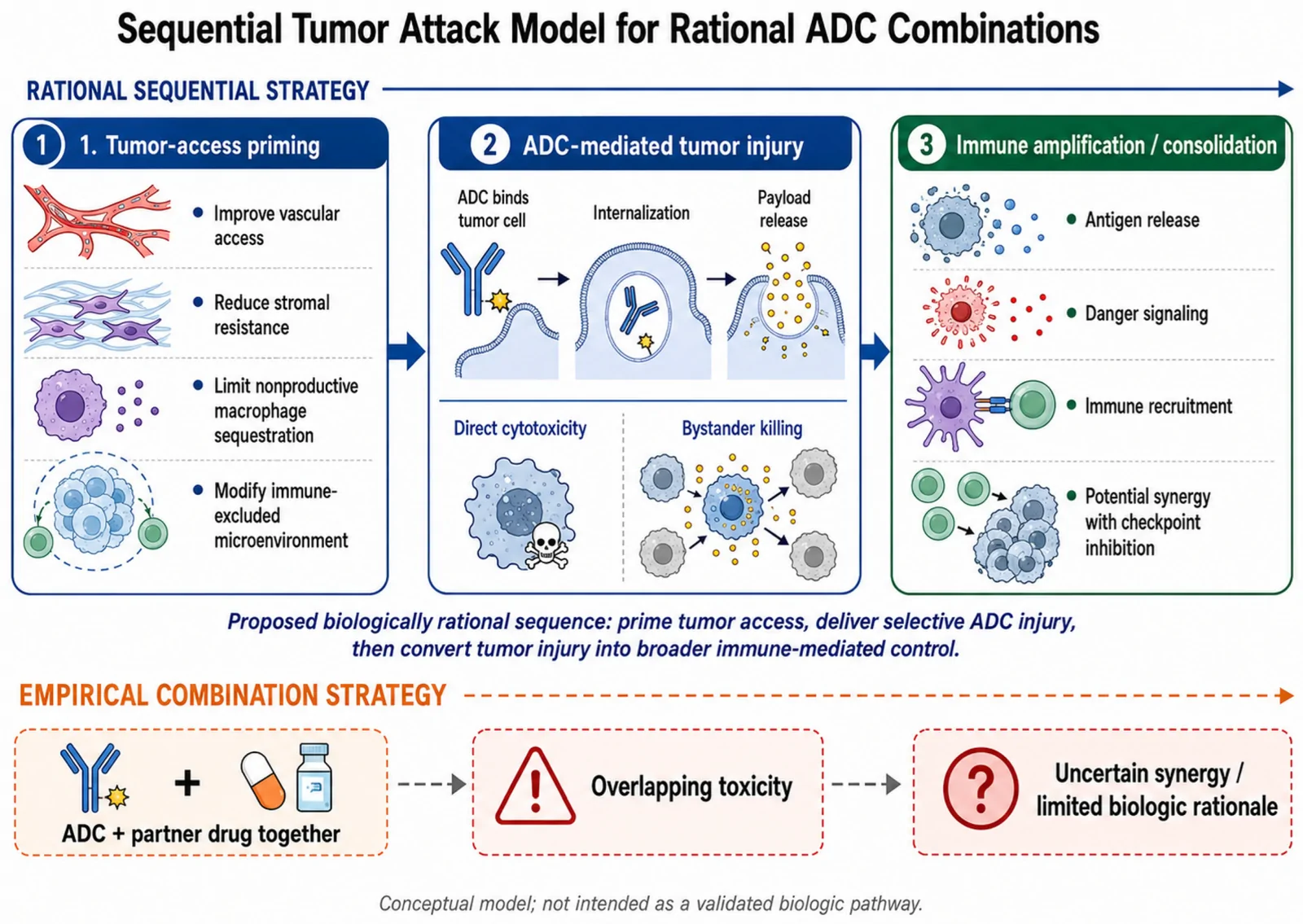
Conceptual Sequential Tumor Attack Model for rational ADC combinations. This hypothesis-generating framework proposes a sequence of tumor-access priming, selective ADC-mediated injury, and immune amplification. Tumor-access priming may improve functional vascular access, reduce stromal barriers or nonproductive macrophage sequestration, and modify an immune-excluded microenvironment. The ADC may then produce direct cytotoxicity and, when the released payload is membrane permeable, bystander killing. ADC-mediated injury may promote antigen release, danger signaling, antigen presentation, and immune recruitment, creating an opportunity for amplification with checkpoint inhibition or other immune-modulating strategies. Individual components are biologically plausible and supported to varying degrees, but the complete sequence remains theoretical and has not been prospectively validated as a clinical paradigm [17,22,47].

This idea is summarized in Figure 1 as a conceptual Sequential Tumor Attack Model. The model proposes, but does not establish, that tumor-access priming could improve delivery, that ADC-mediated injury could generate antigen release and danger signaling, and that immune-directed therapy could amplify the resulting response. It is intended to organize testable hypotheses for future combination studies rather than to define a validated biologic pathway or recommended clinical treatment sequence [17,22,47].

However, the combination literature is advancing faster than the underlying mechanistic certainty. Not every ADC is likely to synergize equally with every partner class. Some combinations may be truly biologically complementary, whereas others may simply layer toxicity without meaningfully improving depth or duration of response. This distinction becomes crucial as ADCs move earlier in therapy, where both efficacy expectations and safety standards are higher [17,19,21,22].

For clinicians and translational investigators, the key challenge is therefore to separate rational combination design from empirical combination expansion. The most valuable future studies will likely be those that define not only whether a combination improves outcomes, but also why it should work in a given biologic context and how that benefit should influence sequencing across the broader treatment course.

### 8.4. Why This Gap Matters

Dose, sequence, and combination represent an inflection point in ADC development because they connect molecular design to real-world therapeutic strategy. A field can tolerate uncertainty about sequencing when only one ADC exists in a late-line setting. It cannot tolerate the same uncertainty once several ADCs compete for use across multiple lines of therapy. In that environment, better engineering alone will not be enough. ADC development must also become more intentional about how these drugs are introduced, ordered, and partnered in clinical practice. Key translational challenges and future directions are summarized in Table 2.

## 9. The Next Wave of Early Development

The next phase of ADC progress will likely depend less on simply adding new antigens and more on addressing the known limitations of current agents.

### 9.1. Site-Specific and Homogeneous Conjugation

Site-specific conjugation aims to reduce product heterogeneity and create more consistent, predictable ADCs. Greater homogeneity may improve pharmacokinetics, reduce unwanted clearance, and provide tighter control over DAR. These properties may be especially important as ADC platforms become more complex [48].

### 9.2. Novel Payloads and Advanced ADC Architectures

Most approved ADCs still rely on a relatively narrow set of payload families. Next-generation programs are exploring a broader range of payloads, including DNA-damaging agents, transcriptional inhibitors, immune-modulating compounds, and other nontraditional warheads. The goal is to widen mechanistic diversity, overcome class-specific resistance, and tailor payload choice more precisely to tumor biology. At the same time, developers are pursuing more sophisticated ADC architectures, including dual-payload and bispecific constructs. Dual-payload ADCs seek to combine complementary mechanisms within a single molecule, potentially addressing tumor heterogeneity and delaying resistance, while bispecific ADCs may enhance selectivity, improve internalization, or enable dual-antigen recognition. Together, these approaches aim to expand the functional capabilities of ADCs beyond conventional single-target, single-payload designs, although they also introduce substantial manufacturing and clinical-development complexity [44,83,84,85].

### 9.3. Masked and Tumor-Activated Platforms

Masked or conditionally activated ADCs are designed to remain relatively inactive in circulation and become activated preferentially in the tumor microenvironment. This approach may widen the therapeutic window, particularly for targets that are limited by normal-tissue expression. It is especially relevant in solid tumors, where truly tumor-exclusive surface antigens are uncommon [86,87].

### 9.4. Immune-Stimulating Conjugates

Another important frontier is the development of immune-stimulating antibody conjugates or related targeted immune-delivery systems. These constructs seek not only to kill tumor cells directly but also to reshape the tumor microenvironment and enhance antitumor immunity. If successful, they could extend ADC principles beyond targeted chemotherapy toward localized immunomodulation [88].

## 10. Regulatory, Pharmacologic, and Manufacturing Considerations

As ADCs become more sophisticated, development is increasingly limited by operational complexity. Manufacturing consistency is essential, particularly for site-specific conjugates, bispecific platforms, and multifunctional constructs. Bioanalytical assessment is also more demanding than for conventional antibodies or small molecules, often requiring separate evaluation of total antibody, conjugated antibody, free payload, and relevant metabolites or catabolites [3,48,49].

Dose optimization remains a major challenge. Traditional oncology development often favors the highest tolerable dose, but ADCs frequently require a more nuanced strategy that balances exposure-response relationships, cumulative toxicity, and delayed organ-specific adverse events. Schedule optimization may be as important as dose level [3,40,49].

Regulatory expectations are also evolving. As the field matures, successful development will increasingly require better companion diagnostics, stronger confirmatory evidence, improved clinical pharmacology characterization, and more careful attention to immunogenicity, drug interactions, and special populations. These trends raise the bar for entry but should ultimately improve the quality and interpretability of ADC development [3,40,49].

## 11. Future Directions and Conclusions

ADCs have evolved into one of the most important therapeutic platforms in contemporary oncology. Their success validates the principle that potent cytotoxic agents can be delivered with sufficient tumor selectivity to produce substantial benefit across multiple malignancies.

Yet the field has also made clear that selectivity is never absolute. Every gain in potency, bystander effect, or target breadth must be balanced against systemic instability, normal-tissue exposure, and the emergence of resistance. The future of ADC therapy will therefore depend less on simple target expansion and more on integrated optimization of chemistry, tumor biology, pharmacology, biomarkers, and clinical strategy.

Perhaps the most important future question is how ADCs should be used once they are no longer isolated products but members of an increasingly crowded therapeutic class. If next-generation platforms are to reach their full potential, the field will need not only better targets and safer payloads, but also a clearer framework for dose optimization, sequential use, and biologically rational combinations.

The next phase of ADC development will be defined not only by new targets and constructs, but by whether the field can establish a mature evidence base for rational dose selection, sequential use, and combination therapy.

## Figures and Tables

**Table 1:** Clinically established antibody–drug conjugates and key design features.

ADC	Target	Payload Class	Linker	Major Disease Settings	Signature Toxicity Considerations
Gemtuzumab ozogamicin [2,5]	CD33	Calicheamicin/DNA damaging	Cleavable	AML	Myelosuppression; hepatic toxicity; VOD/SOS
Brentuximab vedotin [2,5]	CD30	MMAE/microtubule inhibitor	Cleavable	Hodgkin lymphoma; systemic ALCL; CD30-positive lymphomas	Neuropathy; myelosuppression
Trastuzumab emtansine [2,5,16]	HER2	DM1/microtubule inhibitor	Noncleavable	HER2-positive breast cancer	Thrombocytopenia; hepatotoxicity; neuropathy
Inotuzumab ozogamicin [2,5]	CD22	Calicheamicin/DNA damaging	Cleavable	B-cell ALL	VOD/SOS; myelosuppression
Polatuzumab vedotin [2,5,25]	CD79b	MMAE/microtubule inhibitor	Cleavable	DLBCL	Neuropathy; cytopenias; infection risk
Enfortumab vedotin [19,22]	Nectin-4	MMAE/microtubule inhibitor	Cleavable	Urothelial carcinoma	Rash; neuropathy; hyperglycemia
Sacituzumab govitecan [11,26,27]	TROP2	SN-38/topoisomerase I inhibitor	Hydrolysable cleavable	TNBC; HR-positive/HER2-negative breast cancer	Neutropenia; diarrhea
Trastuzumab deruxtecan [16,21,23,24,28,29,30,31,32,33]	HER2	Deruxtecan/topoisomerase I inhibitor	Cleavable	HER2-positive breast cancer; HER2-low/ultralow breast cancer; HER2-positive solid tumors; HER2-mutant NSCLC	ILD/pneumonitis; nausea; myelosuppression
Loncastuximab tesirine [2,5]	CD19	PBD dimer/DNA damaging	Cleavable	Relapsed/refractory large B-cell lymphoma	Edema/effusions; cytopenias; hepatotoxicity
Tisotumab vedotin [9,34]	Tissue factor	MMAE/microtubule inhibitor	Cleavable	Cervical cancer	Ocular toxicity; bleeding; neuropathy
Mirvetuximab soravtansine [20,35]	FRα	DM4/microtubule inhibitor	Cleavable	FRα-positive platinum-resistant ovarian cancer	Ocular toxicity; neuropathy; gastrointestinal toxicity
Datopotamab deruxtecan [12,13,36,37,38]	TROP2	Deruxtecan/topoisomerase I inhibitor	Cleavable	HR-positive/HER2-negative breast cancer; EGFR-mutated NSCLC; selected TNBC settings	Stomatitis; ocular toxicity; ILD/pneumonitis
Telisotuzumab vedotin [14]	c-Met	MMAE/microtubule inhibitor	Cleavable	c-Met-overexpressing nonsquamous NSCLC	Neuropathy; edema; pneumonitis/ILD signal
Belantamab mafodotin [15,39]	BCMA	MMAF/microtubule inhibitor	Noncleavable	Relapsed/refractory multiple myeloma combinations	Keratopathy/ocular toxicity; thrombocytopenia

Note: ADC, antibody–drug conjugate; ALL, acute lymphoblastic leukemia; ALCL, anaplastic large-cell lymphoma; AML, acute myeloid leukemia; BCMA, B-cell maturation antigen; DLBCL, diffuse large B-cell lymphoma; FRα, folate receptor alpha; HER2, human epidermal growth factor receptor 2; HR, hormone receptor; ILD, interstitial lung disease; MMAE, monomethyl auristatin E; MMAF, monomethyl auristatin F; NSCLC, non-small cell lung cancer; PBD, pyrrolobenzodiazepine; TNBC, triple-negative breast cancer; VOD/SOS, veno-occlusive disease/sinusoidal obstruction syndrome.

**Table 2:** Translational challenges and future directions in ADC development.

Translational Challenge	Why It Matters	Examples of Unresolved Questions	Future Direction
Dose optimization [3,40,49]	MTD-based development may not capture delayed or organ-specific ADC toxicity.	Should ADCs be dosed by MTD, exposure-response, payload exposure, or cumulative toxicity?	Prospective dose-optimization cohorts; PK/PD modeling; exposure-toxicity integration.
Biomarker uncertainty [55,56,57,58,59,60]	Static antigen IHC does not fully predict delivery, internalization, bystander effect, or resistance.	Is target expression sufficient? Should metastatic lesions be reassessed?	Spatial biomarkers; quantitative IHC; ctDNA; target reassessment; functional uptake assays.
Sequencing [72,73,74,75]	Multiple ADCs now overlap within breast cancer and other disease spaces.	Is it better to switch target, payload, linker, or all three?	Prospective ADC sequencing trials with paired biopsies and payload-resistance biomarkers.
Cross-resistance [55,72,73,74,75]	Shared payloads may produce resistance even when targets differ.	Can topoisomerase I inhibitor-payload ADCs be used sequentially without loss of efficacy?	Payload-class resistance testing; TOP1 alterations; efflux markers.
Toxicity management [50,51,52,53,54]	ILD, ocular toxicity, neuropathy, and myelosuppression can limit benefit.	Which toxicities require interruption versus surveillance?	Standardized toxicity algorithms; early imaging; ophthalmology co-management.
Tumor penetration [4,47]	Stromal barriers, high interstitial pressure, abnormal vasculature, and macrophage uptake may limit ADC delivery.	How much administered ADC reaches tumor cells versus stroma/macrophages?	Tumor-access priming; stromal modulation; imaging-based ADC delivery assays.
Rational combinations [17,19,21,22]	Empirical combinations may increase toxicity without synergy.	Which partner should be used, and in what sequence?	Biology-driven combinations: priming, ADC injury, then immune amplification.
Next-generation architectures [83,84,85,86,87,88]	Bispecific, dual-payload, masked, and immune-stimulating ADCs may expand the platform.	Which design adds real therapeutic value versus complexity?	Construct-specific clinical validation and biomarker-driven development.

Note: ADC, antibody-drug conjugate; IHC, immunohistochemistry; ILD, interstitial lung disease.

## Data Availability

No new data were created or analyzed in this study. Data sharing is not applicable to this article.

## Abbreviations

**Abbreviation**	**Definition**
ALCL	Anaplastic large-cell lymphoma
ALL	Acute lymphoblastic leukemia
AML	Acute myeloid leukemia
BCMA	B-cell maturation antigen
DAR	Drug-to-antibody ratio
DLBCL	Diffuse large B-cell lymphoma
FRα	Folate receptor alpha
HER2	Human epidermal growth factor receptor 2
HR	Hormone receptor
IHC	Immunohistochemistry
ILD	Interstitial lung disease
MMAE	Monomethyl auristatin E
MMAF	Monomethyl auristatin F
NSCLC	Non-small cell lung cancer
PBD	Pyrrolobenzodiazepine
T-DM1	Trastuzumab emtansine
T-DXd	Trastuzumab deruxtecan
TME	Tumor microenvironment
TNBC	Triple-negative breast cancer
TROP2	Trophoblast cell-surface antigen 2
VOD/SOS	Veno-occlusive disease/sinusoidal obstruction syndrome
